# SIRT1 Is Involved in the Neuroprotection of Pterostilbene Against Amyloid β 25–35-Induced Cognitive Deficits in Mice

**DOI:** 10.3389/fphar.2022.877098

**Published:** 2022-04-14

**Authors:** Lin Zhu, Fangjin Lu, Xiaoran Zhang, Siyuan Liu, Ping Mu

**Affiliations:** ^1^ Department of Biochemistry and Molecular Biology, Shenyang Medical College, Shenyang, China; ^2^ Department of Pharmacology, Shenyang Medical College, Shenyang, China; ^3^ Department of Physiology, Shenyang Medical College, Shenyang, China

**Keywords:** Alzheimer’s disease, pterostilbene, learning–memory, SIRT1, apoptosis

## Abstract

Alzheimer’s disease (AD) is a progressive neurodegenerative disorder characterized by amyloid-β (Aβ) deposits and neurofibrillary tangles. Pterostilbene (PTE), a bioactive component mainly in blueberries, is found to have neuroprotective properties. However, the specific underlying mechanisms of PTE in protecting AD remain unclear. Herein, we explored its effects on Aβ_25–35_-induced neuronal damage *in vivo* and *in vitro* and further compared the roles with its structural analog resveratrol (RES) in improving learning–memory deficits. We found that intragastric administration of PTE (40 mg/kg) displayed more effective neuroprotection on Aβ_25–35_-induced cognitive dysfunction assessed using the novel object test, Y-maze test, and Morris water maze test. Then, we found that PTE improved neuronal plasticity and alleviated neuronal loss both *in vivo* and *in vitro*. Additionally, PTE upregulated the expression of sirtuin-1 (SIRT1) and nuclear factor erythroid 2-related factor 2 (Nrf2) and the level of superoxide dismutase (SOD), and inhibited mitochondria-dependent apoptosis in the Aβ_25–35_-treated group. However, SIRT1 inhibitor EX527 reversed the neuroprotection and induced a drop in mitochondrial membrane potential in PTE-treated primary cortical neurons. Our data suggest that PTE’s enhancing learning–memory ability and improving neuroplasticity might be related to inhibiting mitochondria-dependent apoptosis via the antioxidant effect regulated by SIRT1/Nrf2 in AD.

## Introduction

The World Alzheimer Report 2019 shows that over 50 million people worldwide are living with dementia and the number will be estimated to increase to 152 million by 2050. Meanwhile, the expected annual cost of dementia in the United States is $1 trillion. Alzheimer’s disease (AD), one of the most common dementia in older adults, is a devastating neurodegenerative disease characterized by the accumulation of Aβ plaques, neurofibrillary tangles, and severe neuronal loss (Winblad et al., 2016). Progressive cognitive impairment and memory loss are the classical hallmarks of AD (Shoji, 2014). In the earliest stage of AD, the primary cause of the cognitive deficits is thought to be the synaptic loss induced by Aβ, and the synaptic plasticity is directly related to learning–memory processes (Flores-Munoz et al., 2020).

Mitochondria play an essential role in cellular physiology by producing adenosine triphosphate (ATP) and clearance of reactive oxygen species (Nunnari and Suomalainen, 2012). Mitochondrial dysfunction has been well accepted as a critical target in neurodegenerative disease, which is increasingly recognized as one of the prime factors in the progression of AD (Butterfield and Halliwell, 2019). Mitochondrial damage, such as membrane potential loss, cristae disorder, and imbalanced fusion–fission status, was observed in AD models (Swerdlow, 2018). Reports indicate that Aβ-induced mitochondrial dysfunction contributes to injury to the structure and function of synapses, finally leading to learning–memory deficits (Mattson et al., 2008; Fanibunda et al., 2019), and the mechanism of injury may be to induce neuronal apoptosis (Fang et al., 2021). The regulation of mitochondrial function by activating SIRT1 has recently emerged as an essential pathway in regulating AD. SIRT1 is a nicotinamide adenosine dinucleotide-dependent protein deacetylase and serves as a master regulator of mitochondrial function through downstream pathways including SOD modulation (Min et al., 2018; Zhao et al., 2020). SIRT1 may produce a neuroprotective effect by enhancing the activity of Nrf2, which eventually increases SOD activity (Liu et al., 2017; Zhu et al., 2021). It has been reported that SIRT1 expression is decreased in the brain regions of AD patients and AD mice (Lutz et al., 2014; Corpas et al., 2017). Meanwhile, overexpressed SIRT1 enhances neurite outgrowth and dendritic complexity to protect neuronal survival against Aβ_1–42_ (Guo et al., 2011).

Pterostilbene (3,5-dimethoxy-4′-hydroxystilbene, PTE), a natural stilbenoid, is mainly found in grapes, berries, and *Pterocarpus marsupium* heartwood. The production of PTE in high concentrations has been found in the Xarello grape variety and in low concentrations in the leaf extracts of other grape varieties (Douillet-Breuil et al., 1999). Research also shows that the content of PTE in blueberries can reach almost 520 ng/g dry sample (Rimando et al., 2004). As a dimethylated analog of resveratrol (trans-3, 5, 4′-trihydroxystilbene, RES), it has been proved that PTE possesses high bioavailability for possible broad activities, such as antioxidant, anticancer, and neuroprotective properties (Poulose et al., 2015; Liu J. et al., 2020). RES is a potent activator of SIRT1, which has been found to protect neurons through the attenuation of Aβ-induced toxicity in AD (Rege et al., 2014). Reports have provided evidence that neuroprotective properties of RES in AD are due to improved mitochondrial dysfunction and increased anti-inflammatory and antioxidant properties (Sawda et al., 2017; Vaiserman et al., 2019; Shaito et al., 2020). However, the exact underlying mechanism of PTE in improving Aβ_25–35_-induced neuroplastic injury remains to be elucidated.

Therefore, in the current study, we established the AD models with Aβ_25–35_-induced mice and primary neurons to explore the protective mechanism of PTE in cognitive functions.

## Materials and Methods

### Materials

PTE (Figure 2A) and RES (Figure 2B), purchased from Dalian Meilun Biotechnology (CHN), respectively, were dissolved in saline with 0.1% dimethyl sulfoxide (DMSO). Aβ_25–35_, purchased from Sigma-Aldrich (United States), was dissolved in saline at a concentration of 3 mM and incubated at 37 °C for 5 days to induce the formation of aggregated Aβ_25–35_ (Zhu et al., 2021). All other chemical reagents used in this study were of analytical grade.

### Animals

Male KM mice (4 week-old) were purchased from Liaoning Changsheng Biotechnology Co. Ltd., and kept on a 12-h light–dark cycle in a temperature-controlled room at 20 ± 2°C with a relative humidity of 55 ± 5%. The mice were allowed *ad libitum* access to food and water.

### Experimental Design

**FIGURE 1:**
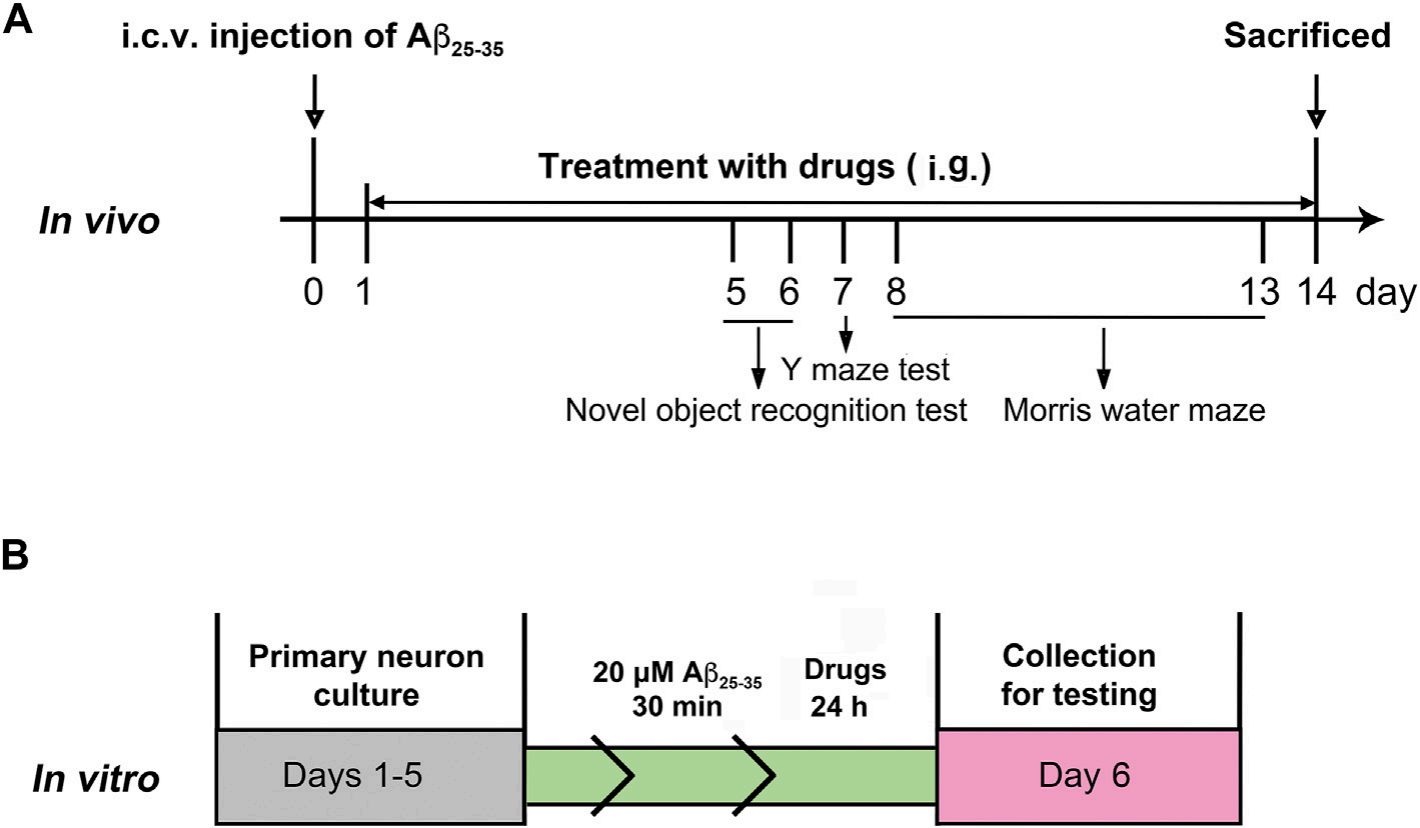
Experimental protocols *in vivo* **(A)** and *in vitro* **(B)**.

After a week of adaptation, the mice received a single intracerebroventricular injection (i.c.v.) of Aβ_25–35_ (9 nmol/3 μl), and the sham-operated animals underwent the same surgery with the infusion of saline (Maurice et al., 1996). After 24 h, the mice were subjected to intragastric administration of PTE (10 or 40 mg/kg/d) or RES (40 mg/kg/d) until the mice were killed. Behavioral tests were started on day 5 after injection. The experimental procedure is schematically represented in Figure 1A.

### Behavioral Analyses

#### Novel Object Test

The experiment consisting of habituation, training, and testing phases was performed following the previous protocol (Vogel-Ciernia and Wood, 2014). For the habituation session (day 5), the animals were habituated individually to an open field arena. The mice were placed at the center of the open field arena (40 cm × 40 cm × 35 cm) for 5 min twice a day under dim overhead lighting conditions. For the training session (day 6), two identical objects (A1 and A2) were placed in the open field (10 cm from the back wall), and the mice were singly placed at the center of the opposite wall. The animals were allowed to explore each object freely for 5 min. The testing phase was performed 1 h after the training session. The animal explored the open field arena for 5 min in the presence of one familiar and one novel object with different sizes, shapes, and colors (A1 and B). The arena and objects were cleaned with 75% (v/v) ethyl alcohol. A mouse was scored as exploring an object when its head was oriented toward the object within a distance of 1 cm or when the nose was touching the object. The relative exploration time was recorded and expressed by a discrimination index (DI):

$$DI=\frac{tA2\;–\;tA1}{tA1\;+\;tA2}\times 100\%\;\text{ or}\;\frac{tB\;–\;tA1}{tA1\;+\;tB}\times 100\%,$$

where t represents time.

#### Y-Maze Test

Spontaneous alternation behavior was evaluating spatial working memory performance in the Y-maze test (Swonger and Rech, 1972). A custom-made Y-maze with three identical arms (40 × 10 × 12 cm, 120° apart) was placed under dim lighting conditions. Each mouse was placed at the end of one fixed arm facing the wall and allowed to explore the maze freely for 5 min. The total number of arm entries (N) and the sequence of entries were recorded. Successful alternation was defined as consecutive entry into all three arms. Percent alternation was calculated as the number of successful alternations/(N-2) × 100%.

#### Spatial Learning

The Morris water maze task (days 8–13) was used to assess spatial memory performance (Morris et al., 1982). Briefly, during the training session, the mice have been trained on the Morris water maze two trials per day for five consecutive days with a black circular pool (90 cm in diameter), and a circular platform (10 cm in diameter) was submerged 1 cm beneath the surface of the water. The pool temperature was maintained at 23 ± 1°C to avoid hypothermia, and the platform was placed in the middle of one quadrant throughout the training session. The mice were allowed to search for the hidden platform within 60 s. The probe test was conducted on the sixth day. For probe trials, the platform was removed from the pool, and the mice were allowed to swim freely for 60 s. Each test was recorded with a digital camera, and the activity analysis was performed with the behavioral tracking system (JiLiang ShangHai, CHN).

### Immunohistochemistry Staining

The mice were anesthetized with 20% urethane (10 ml/kg, i.p.). The brains were fixed in 4% paraformaldehyde overnight and then dehydrated in 20 and 30% sucrose until they sunk. The tissues were cut into 15-μm-thick coronal sections. The sections were stained with neuronal nuclear protein (NeuN) antibody (1:400, CST, United States) overnight at 4 °C and incubated with an anti-rabbit secondary antibody (1:500, Invitrogen, United States) for 2 h at room temperature. Images were captured by using the Nikon microscope (Eclipse Ci-E, JP).

### Transmission Electron Microscopy Analysis

TEM was performed with some modifications, as previously described (Zhu et al., 2021). The tissues were fixed with 2.5% glutaraldehyde dissolved in 0.01 M phosphate-buffered saline (PBS) overnight at 4°C. After removing and post-fixing by immersion in the fixative overnight at 4°C, the samples were treated with 1% osmium tetroxide for 3 h, dehydrated with graded ethanol and acetone and embedded in resin. Then, the sections were stained with 4% uranyl acetate and 0.5% citrate. The ultrastructure, especially the neuronal nuclei, synaptic, and mitochondrial structure of the parietal cortex and hippocampal CA1, was detected and the images were taken by using the transmission electron microscope (JEOL H-7650, JP).

### Western Blotting

Brain tissue or primary neuronal cells were homogenized in ice-cold lysis buffer with protease inhibitors and phosphatase inhibitors, and the protein concentrations were determined by the BCA assay (Beyotime Biotechnology, CHN). The samples were subjected to sodium dodecyl sulfate–polyacrylamide gel electrophoresis (SDS-PAGE). Briefly, equal amounts (20–30 μg) of proteins were loaded per lane, transferred to 0.45-μm polyvinylidene difluoride (PVDF) membrane, and blocked for 2 h at room temperature. Afterward, the membranes were incubated overnight at 4°C with primary antibodies: postsynaptic density protein 95 (PSD-95) (1:1000, CST, United States), synapsin-1 (SYN-1) (1:1200, CST, USA), NeuN (1:1000, CST, United States), SIRT1 (1:1000, Abcam, UK), Nrf2 (1:1000, Abcam, UK), Bcl-2-associated X protein (Bax) (1:4000, Proteintech, United States), B-cell lymphoma 2 (Bcl-2) (1:800, Proteintech, United States), and β-actin (1:400, Santa Cruz, United States). Then, the membranes were incubated with secondary antibodies (1:10,000, Jackson ImmunoResearch, United States). Protein bands were visualized with enhanced chemiluminescence (ECL). Then, the intensity of the bands was quantified by ImageJ software (version 1.8.8, National Institutes of Health, United States) and corrected with the corresponding β-actin level. The results were expressed as values normalized to the loading sham.

### Measurement of Total Superoxide Dismutase Activity

The tissues were lysed with a lysate buffer and then centrifuged at 12,000 g at 4°C for 5 min. Afterward, an aliquot of the supernatant was used for detection. Superoxide dismutase enzyme activities were measured using a commercial SOD Assay Kit-WST (Beyotime Biotechnology, CHN) in accordance with the manufacturer’s protocol. The values were recorded using a microplate reader (CLARIOstar, BMG LABTECH, GER). The concentration of total proteins in the cells was quantified by using the BCA Kit (Takara, JP).

### Primary Neuronal Cultures

The cerebral cortical primary neurons were obtained from newborn KM mice within 24 h as per previously described procedures (Zhu et al., 2018). The neurons were plated onto 96-well dishes or glass slides before coating with poly-l-lysine. The cultures were maintained in Neurobasal/B27 medium, which was changed every 2–3 days. After culturing for 5 days, the primary cortical neurons were treated with aggregated Aβ_25–35_ (20 μM) and drugs for 24 h, as shown in Figure 1B.

### Cell Viability Assay

The viability of the primary cortical neurons was evaluated by using a Cell Counting Kit-8 (CCK-8) (Vazyne Biotech, CHN) following the manufacturer’s protocol. The neurons were (1 × 10^6^/well) cultured in 96-well plates. After culturing for 5 days, the neurons were pretreated with Aβ_25–35_ (20 μM) for 30 min and then incubated with PTE (0.5, 2, or 10 μM) for 24 h at 37°C. Subsequently, 10 μL CCK-8 solution was added into each well for an additional 2 h. The viability was detected by using a microplate reader (CLARIOstar, BMG LABTECH, GER).

### Immunofluorescence

The neurons grown on poly-l-lysine-coated glass slides were fixed with 4% paraformaldehyde solution in PBS for 20 min and permeabilized with 0.3% Triton-X100 in PBS for 10 min at room temperature. Then, the neurons were incubated with primary antibody microtubule-associated protein 2 (MAP-2) (1:200, Santa Cruz, United States) overnight at 4°C. After washing with PBS, the neurons were incubated with an anti-mouse secondary antibody (1:500, Invitrogen, United States) for 2 h at room temperature. Images were captured by using the Leica fluorescence microscope (DMI8, GER), and the neurons in the images were analysis by ImageJ software.

### JC-1 Fluorescence Analysis

JC-1 is a membrane-permeable lipophilic dye that accumulates in the mitochondria. JC-1 exists as J-monomers in the cytoplasm (green fluorescence) and aggregate to form J-aggregates in the mitochondrial matrix (red fluorescence). Mitochondrial depolarization can be measured as an increasing green/red fluorescent intensity ratio to detect neuronal early apoptosis (Brooks et al., 2013).

Neurons were stained with the JC-1 dye (Beyotime Biotechnology, CHN) to detect the mitochondrial membrane potential. The neurons were treated with JC-1 working fluid for 20 min at 37°C. Then, the stained neurons were rinsed twice using JC-1 staining buffer, and a fresh medium was added. Images were captured by using the Nikon microscope (Ts2, JP). ImageJ software was used to for fluorescence intensity analysis.

### Statistical Analysis

All statistical analyses were carried out using SPSS 26.0 software (SPSS Inc, Chicago, IL, United States). The data were analyzed using one-way analysis of variance (ANOVA), followed by the LSD multiple comparisons test with homogeneity or Dunnett’s T3 test with heterogeneity of variance. The data were expressed as mean ± SD. *p* < 0.05 was considered statistically significant.

## Results

### Pterostilbene and Resveratrol Increased the Protein Expression of Sirtuin-1 in Alzheimer’s Disease Mice

**FIGURE 2:**
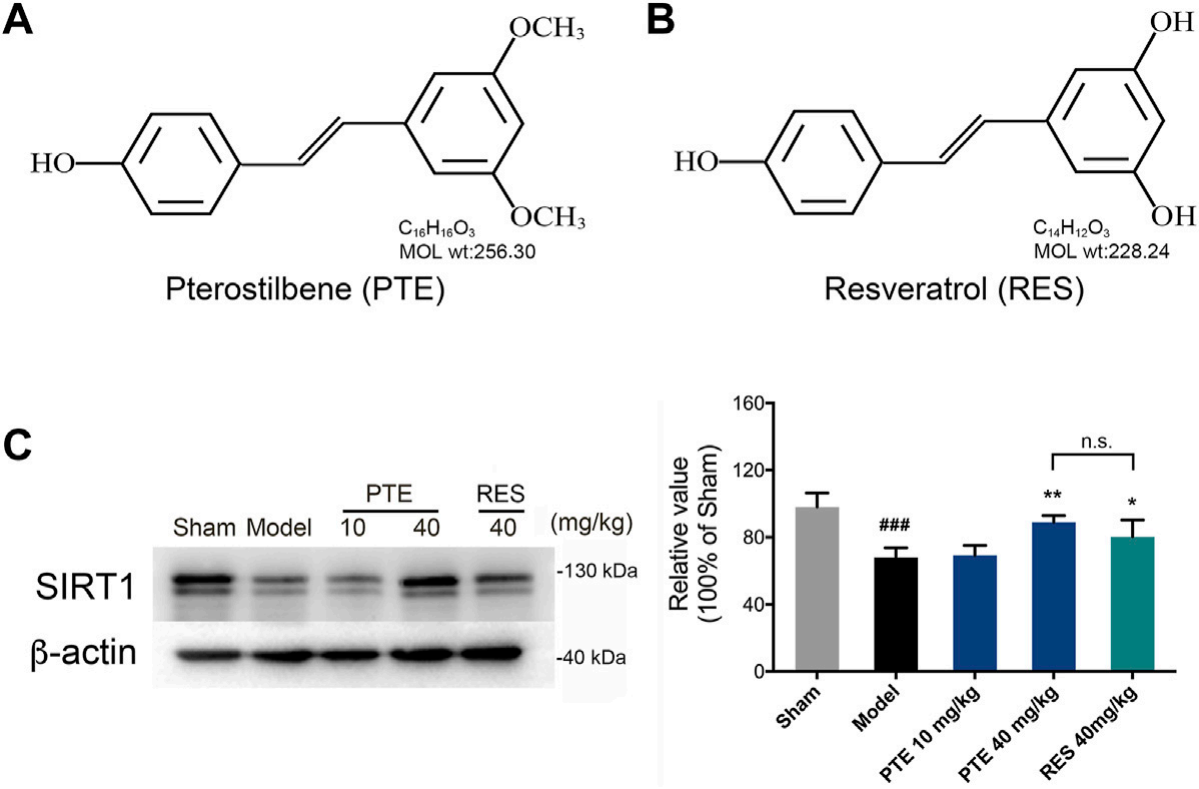
Effects of PTE and RES on the expression of SIRT1 in AD mice. **(A-B)** Chemical structures of PTE **(A)** and RES **(B)**, respectively. **(C)** Western blotting band and quantitative analysis of SIRT1 in the cerebral cortex of mice. Data are shown as mean ± SD (n = 3–4). ^###^ *p* < 0.001 *vs* sham; ^*^ *p* < 0.05, ^**^ *p* < 0.01 *vs* model.

PTE has a chemical structure similar to that of RES, as shown in Figures 2A,B. Studies have demonstrated that treatment with RES exerts neuroprotective effects depending on SIRT1 in AD (Martin, 2017; Gomes et al., 2018). To detect the role of PTE, we detected the expression of SIRT1 in AD mice. The results showed that treatment with PTE and RES both increased the expression of SIRT1, whereas no significant difference between PTE (40 mg/kg) and RES (Figure 2C). These results indicate that PTE is a similar compound to RES and probably has the effect of regulating SIRT1 to improve learning–memory deficits in AD mice.

### Pterostilbene Was More Potent in Improving Learning–Memory Deficits in Alzheimer’s Disease Mice

**FIGURE 3:**
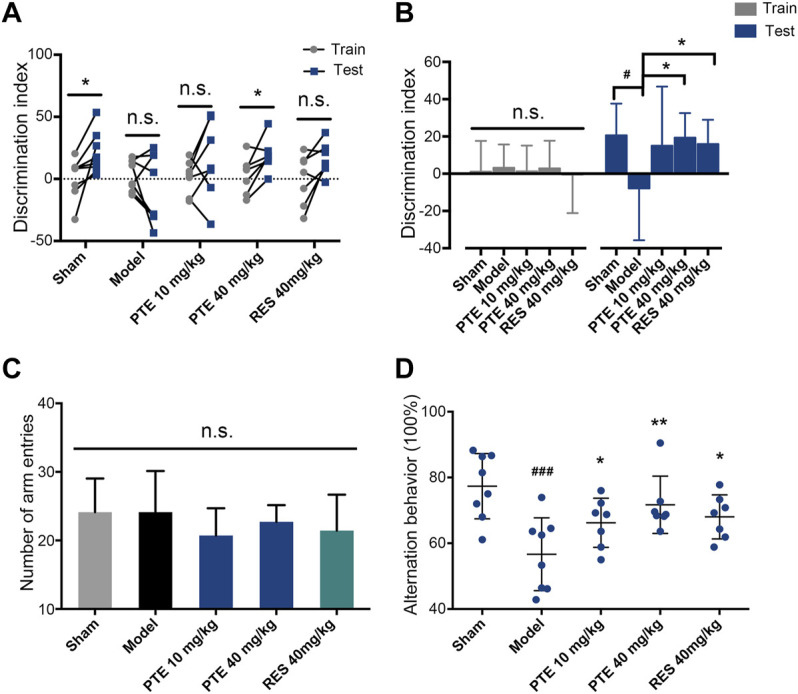
Effects of PTE and RES on the novel object test and Y-maze test in AD mice. **(A–B)** Discrimination index of each mouse **(A)** and the average discrimination index of each group **(B)** during training and testing periods in the novel object test. **(C–D)** The total number of arm entries **(C)** and spontaneous alternation behavior **(D)** in the Y-maze test. Data are shown as mean ± SD (n = 7–8). ^#^ *p* < 0.05, ^###^ *p* < 0.001 *vs* sham; ^*^ *p* < 0.05, ^**^ *p* < 0.01 *vs* model.

**FIGURE 4:**
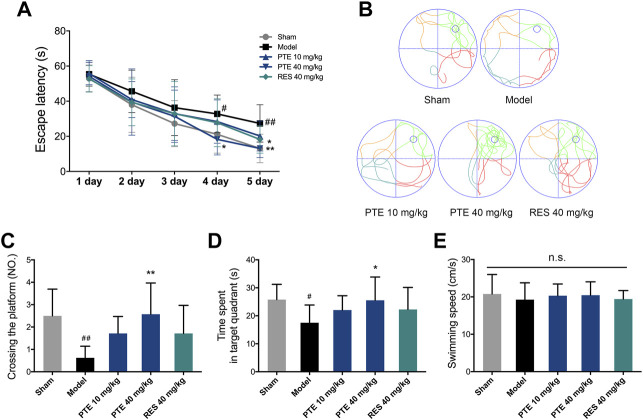
Effects of PTE and RES on Morris water maze test in AD mice. **(A)** Escape latency in the Morris water maze test during the training period. **(B)** Typical swimming tracks of mice in the probe test. **(C–E)** Quantification of the number of crossing the platform, exploring time in the target quadrant, and swimming speed during the probe test. Data are shown as mean ± SD (n = 7–8). ^#^ *p* < 0.05, ^##^ *p* < 0.01 *vs* sham; ^*^ *p* < 0.05, ^**^ *p* < 0.01 *vs* model.

To assess the effects of PTE and RES on learning–memory, we performed the behavioral tests. First, the imaginal memory was evaluated in this study using the novel object test. The results revealed that PTE-treated mice showed more interest in the novel object and spent more time exploring the novel object than the previously studied object (Figure 3A). In the training phase, there was no significant difference in the time of exploring the two identical objects between groups, while both PTE and RES treatments exhibited an increased exploration time to explore the novel object in the testing phase (Figure 3B). Then, we detected the working memory ability in the Y-maze test. The total number of arm entries is shown in Figure 3C, and no significant differences were found among each group (Figure 3C). However, both PTE and RES treatment significantly increased spontaneous alternation behavior (Figure 3D). To investigate the effects of PTE and RES on spatial learning–memory, we used the Morris water maze test in the study. During the training session, compared with the model group, the PTE-treated mice spent significantly less time to locate the platform on the fourth day and the RES group on the fifth day (Figure 4A). Representative pictures of the path chart in the probe trial of the Morris water maze are shown in Figure 4B. In the probe test on the 6th day, treatment with PTE notably increased the number of crossing the platform and the time spent in the target quadrant but had no significant differences in the swimming speed (Figures 4C–E). These results suggest that PTE could more comprehensively improve learning–memory deficits in AD mice.

### Pterostilbene Improved Synaptic Plasticity and Mitochondrial Injury in Alzheimer’s Disease Mice

**FIGURE 5:**
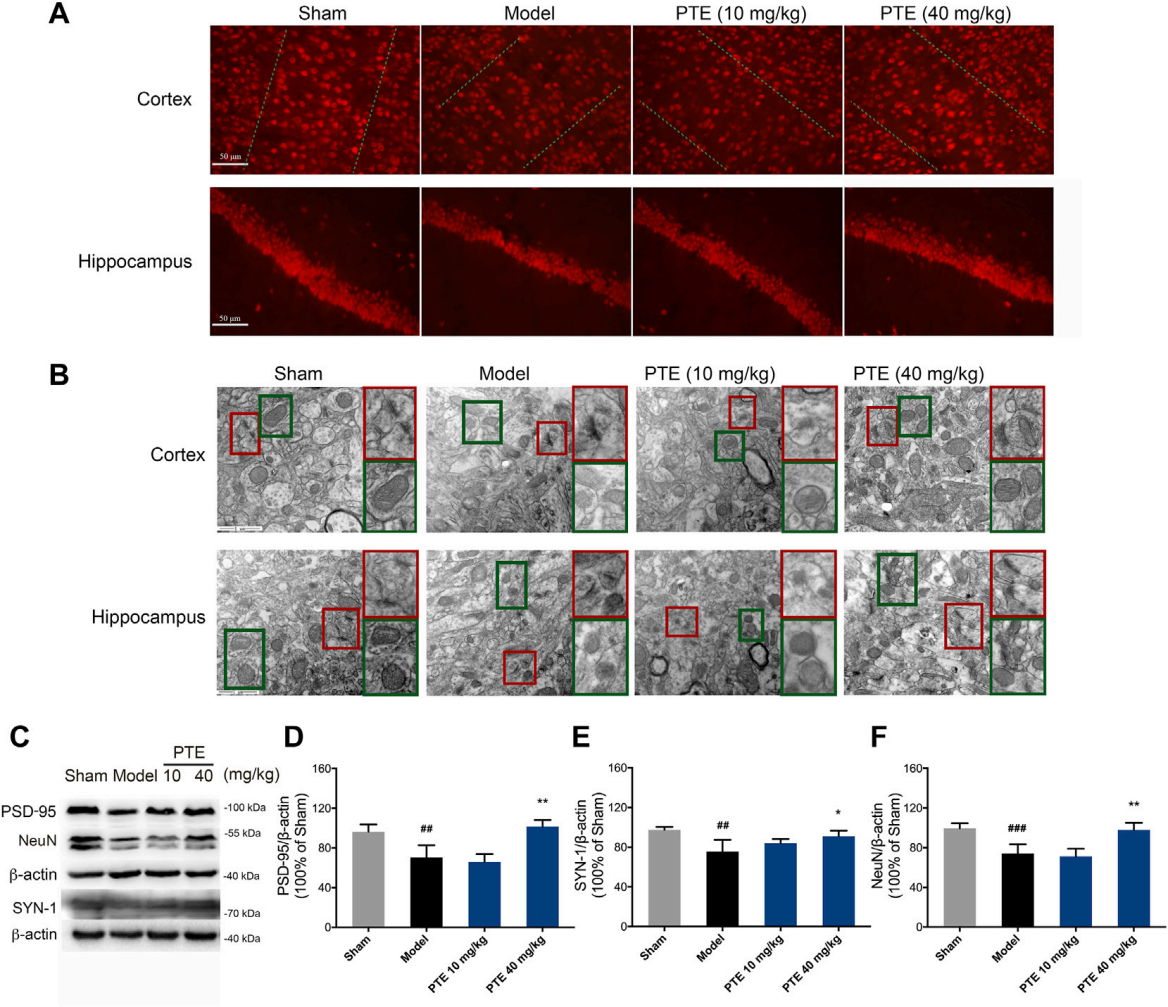
Effects of PTE on neuronal and mitochondrial injury in AD mice. **(A)** NeuN-positive cells in the cerebral cortex and hippocampus were detected by immunofluorescence. **(B)** Representative transmission electron microscopy images showing the synaptic (red square) and mitochondrial structures (green square) in the cerebral cortex and hippocampus. **(C–F)** Western blotting bands **(C)** and quantitative analysis of synaptic plasticity-related proteins PSD-95 **(D)**, SYN-1 **(E)**, and NeuN **(F)** in the cerebral cortex of mice. Data are shown as mean ± SD (n = 3–4). ^##^ *p* < 0.01, ^###^ *p* < 0.001 *vs* sham; ^*^ *p* < 0.05, ^**^ *p* < 0.01 *vs* model.

It is well known that neurons and their synaptic structures are the basis of learning and memory. Herein, the PTE-treated mice exhibited an increase in the expression of NeuN-positive cells both in the cerebral cortex and hippocampus compared with the model mice (Figure 5A). The synaptic structure is composed of the presynaptic membrane, synaptic gap, and postsynaptic membrane. We used transmission electron microscopy to examine the ultrastructure of the cerebral cortex and hippocampal CA1 region in mice. The results showed that PTE improved ambiguous synaptic structure compared with the model mice (Figure 5B). In addition, protein analysis revealed that neuronal proteins NeuN, PSD-95, and SYN-1 were significantly increased after treatment with PTE (Figures 5C–F). The results also found that PTE administration improved mitochondrial crest impairment (Figure 5B). These data indicate that treatment with PTE could ameliorate neuronal and mitochondrial injury in AD mice.

### Pterostilbene Increased Antioxidant Effect and Inhibited Neuronal Apoptosis in Alzheimer’s Disease Mice

**FIGURE 6:**
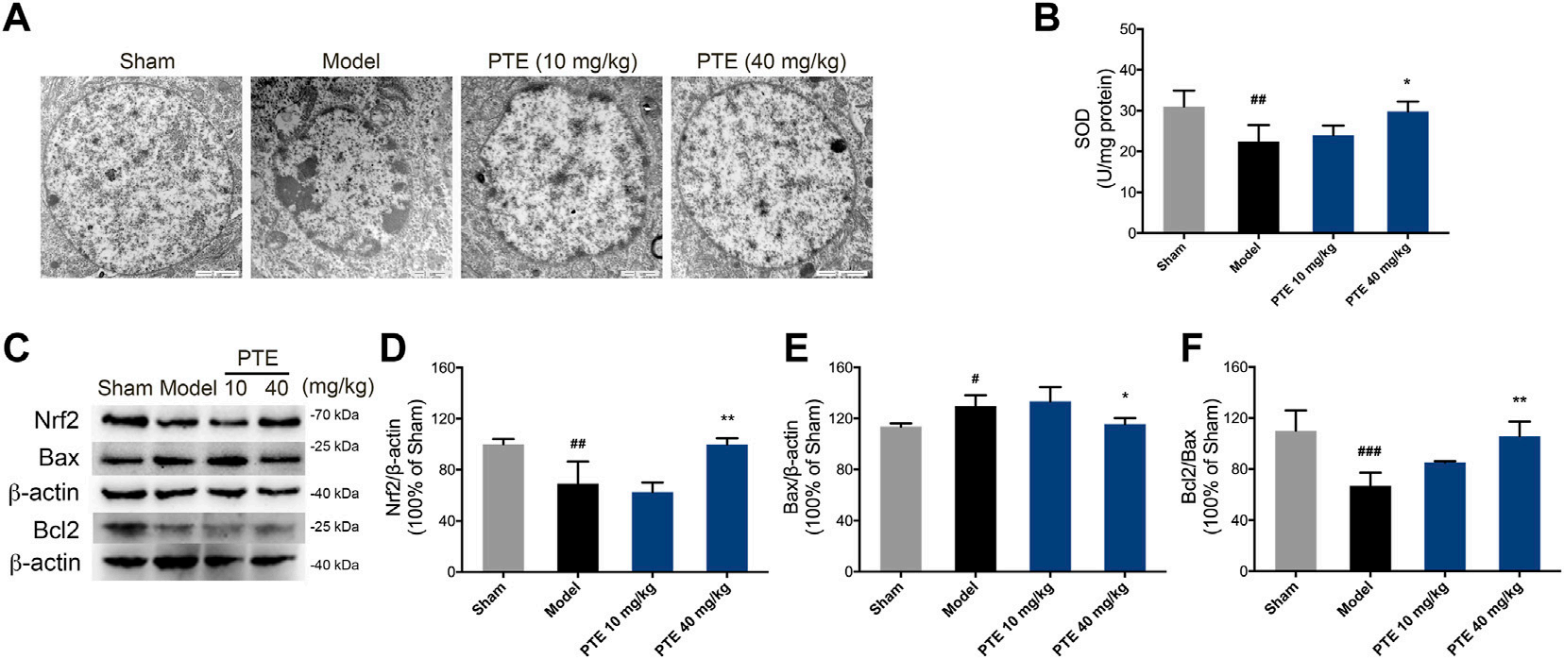
Effects of PTE on SIRT1/Nrf2-induced antioxidant and antiapoptosis in AD mice. **(A)** Electron microscopy images of neuronal nuclei in the cerebral cortex. **(B)** The level of SOD was detected in the cerebral cortex. **(C–F)** Western blotting bands **(C)** and quantitative analysis of Nrf2 **(D)**, Bax **(E)**, and Bcl2/Bax **(F)**, respectively. Data are shown as mean ± SD (n = 3–4). ^#^ *p* < 0.05, ^##^ *p* < 0.01, ^###^ *p* < 0.001 *vs* sham; ^*^ *p* < 0.05, ^**^ *p* < 0.01 *vs* model.

To evaluate the protective effect of PTE on mitochondrial function, the level of apoptosis was determined. In this study, model mice showed chromatin condensation and chromatin accumulation along the inside of the nuclear membrane, while model mice treated with PTE displayed comparatively complete nuclear structures (Figure 6A). We further found that mitochondria-dependent apoptotic protein Bax was decreased, and Bcl2/Bax was increased in PTE-treated mice compared with model mice (Figures 6C,E,F).

The SIRT1/Nrf2 antioxidant pathway was introduced to elucidate the underlying mechanism of PTE in apoptosis caused by mitochondrial damage. The results showed that the expression of SIRT1 (Figure 2C) and Nrf2 (Figures 6C,D) was significantly increased in PTE-treated mice compared with the model mice. Meanwhile the level of SOD was also increased after treatment with PTE (Figure 6B). These results suggest that PTE administration modulates the SIRT1/Nrf2-related antioxidant effect and inhibits neuronal apoptosis to protect neurons in AD mice.

### Pterostilbene Protected Primary Neurons Against Aβ_25–35_-Induced Neuronal and Mitochondrial Injury

**FIGURE 7:**
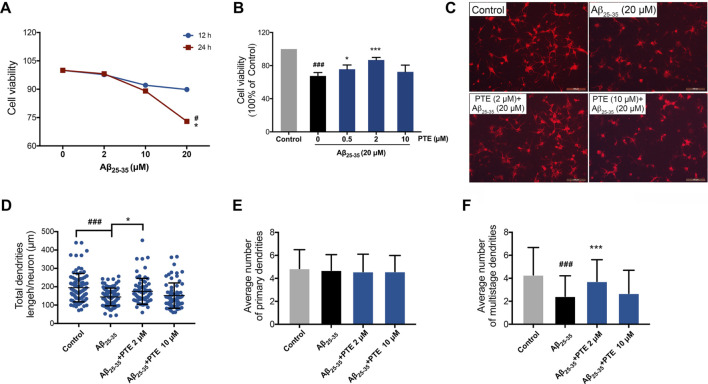
Effects of PTE on Aβ_25-35_-induced neuronal and mitochondrial injury. **(A)** Cell viability of Aβ_25-35_ on neurons was determined by CCK-8 assay. ^#^ *p* < 0.05 *vs* neurons treated without Aβ_25-35_; ^*^ *p* < 0.05 *vs* neurons treated with 2 μM Aβ_25-35_ for 24 h. **(B)** Cell viability determination of Aβ_25-35_-induced neurons after treatment with PTE. **(C)** Neuronal morphology of dendrites was determined with MAP-2 immunofluorescence (70–90 cortical neurons per group were calculated). **(D–F)** Quantifications of the total dendritic length **(D)**, the average number of primary dendrites **(E)**, and multistage dendrites per neuron **(F)**. Data are shown as mean ± SD (n = 3). ^###^ *p* < 0.001 *vs* control; ^*^ *p* < 0.05, ^***^ *p* < 0.001 *vs* Aβ_25-35_ group.

To further investigate the protective effect of PTE against Aβ_25–35_-induced neuronal impairment, primary cortical neurons extracted from mice born within 24 h were used *in vitro* study. Herein, we found that 20 μM Aβ_25–35_ treated for 24 h showed a significant decrease in neuronal viability (Figure 7A), whereas PTE at the concentration of 0.5 and 2 μM both significantly increased the neuronal viability against Aβ_25–35_-induced neuronal damage (Figure 7B). In the following experiments, the concentration of 2 μM of PTE was used to investigate the neuroprotection. We observed that PTE (2 μM) could improve the neuronal structure (Figure 7C). Statistical results showed that the primary dendrites of neurons had no difference in each group (Figure 7E), while when compared with the administration of Aβ_25–35_, the neurons treated with PTE could significantly increase the total dendrite length (Figure 7D) and the number of multistage dendrites (Figure 7F). These results suggest that PTE exerts protective effects to maintain the neuronal structure in primary cortical neurons.

### Pterostilbene Reduced Mitochondria-Dependent Apoptosis of Primary Neurons Was Inhibited by EX527

**FIGURE 8:**
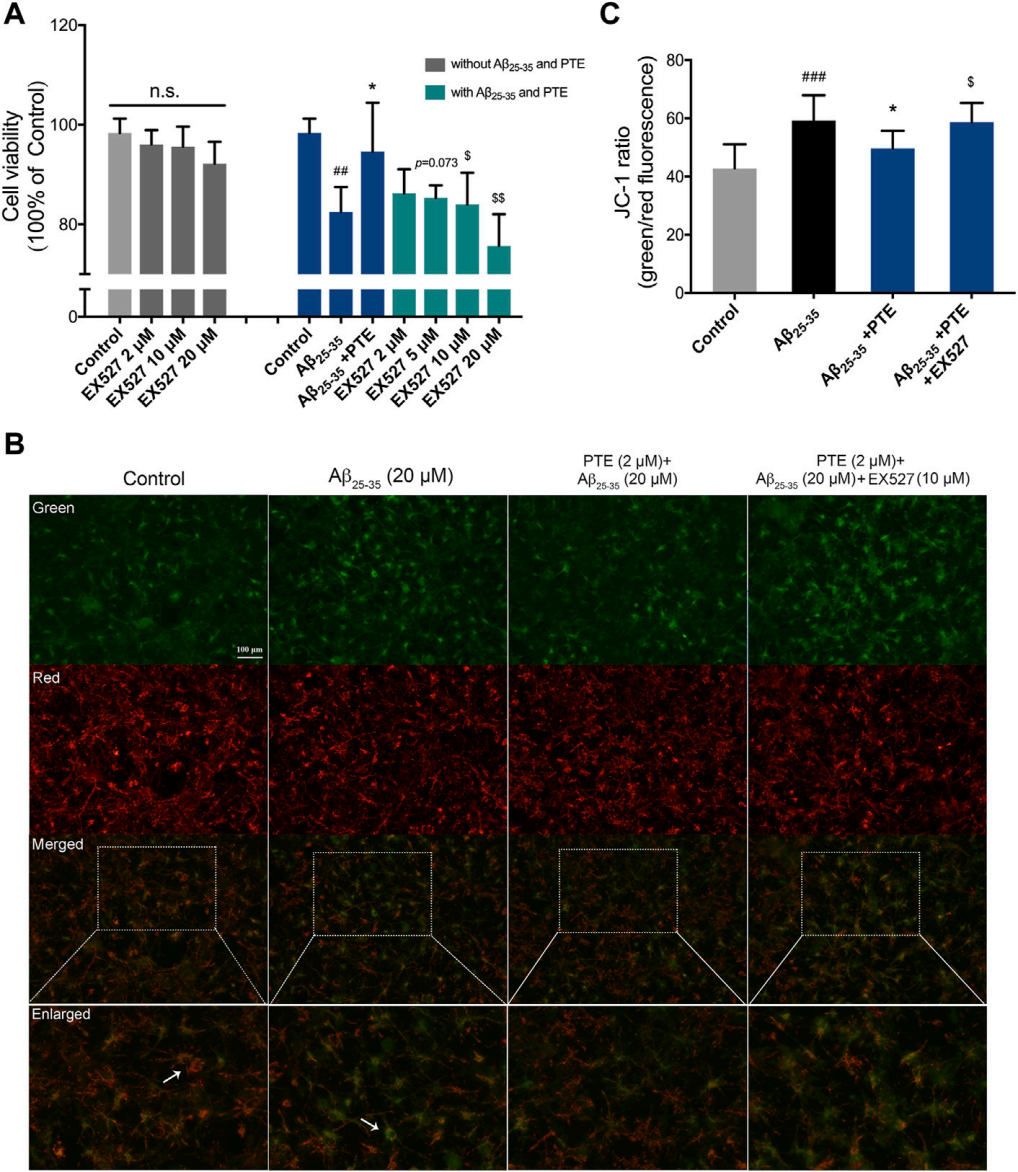
Effects of EX527 on neuronal apoptosis after treatment with PTE. **(A)** Cell viability on neurons after treatment with EX527 was determined. **(B)** Mitochondrial membrane potential was detected by JC-1 dye (green for J-monomers; red for J-aggregates). **(C)** Ratio of green/red fluorescence intensity in each group. Data are shown as mean ± SD (n = 3). ^##^ *p* < 0.01, ^###^ *p* < 0.001 *vs* control; ^*^ *p* < 0.05 *vs* Aβ_25-35_ group; ^$^ *p* < 0.05, ^$$^ *p* < 0.01 *vs* Aβ_25-35_ + PTE group.

In order to investigate whether the antiapoptotic effect of PTE is regulated by the SIRT1/Nrf2 antioxidant pathway, SIRT1 inhibitor EX527 was used in the study. The results showed that treatment with EX527 alone for 24 h did not affect neuronal viability (Figure 8A). PTE reduced neuronal damage caused by Aβ_25–35_, and this protective effect of PTE was blocked by EX527 (Figure 8A). Further research found that primary cortical neurons treated with PTE could attenuate the ratio of green/red fluorescence intensity to stabilize mitochondrial membrane potential compared with the Aβ_25–35_ group (Figures 8B,C). However, EX527 reversed the antiapoptotic effect of PTE on stabilizing mitochondrial membrane potential (Figures 8B,C). Collectively, these data indicate that PTE could produce an antiapoptotic effect to protect neurons by regulating SIRT1.

## Discussion

The process of AD is always accompanied by the loss of learning–memory and the pathological changes of brain nerves (Zhu et al., 2018). Curing or slowing the occurrence and development of AD and improving the patients’ quality of life have always been the goals pursued by scientific researchers. The accumulation of Aβ is one of the pathogenic factors in AD. Aβ_25–35_ is a smaller fragment with 11 amino acids less than the full-length peptide, retaining most of the toxicological properties of Aβ_1–42_ (Pike et al., 1991). In this study, we used the Aβ_25–35_-induced experimental model of AD *in vivo* and *in vitro* to investigate the pharmacological effects of PTE on the nervous system.

Among stilbenes (RES, piceid, viniferins, etc.), RES is the most relevant compound due to its well-known bioactivity. *In vivo* and *in vitro* studies show that RES can prevent or reduce a wide range of diseases, such as neurodegenerative diseases (Zhao et al., 2015; Broderick et al., 2020). RES has potential neuroprotective roles in the treatment of moderate to mild AD (Gu et al., 2021). However, confirmation of RES in humans is very limited, and it fails to show efficacy in clinical assays (Turner et al., 2015; Gu et al., 2021). It may be due to its own rapid metabolism and the differences in diet and interindividual gut microbiota, resulting in low bioavailability of RES and affecting production of RES metabolites (Tomé-Carneiro et al., 2013). Overcoming the poor bioavailability and finding the effective structural analogs of RES may be a direction for the subsequent drug development.

PTE, a natural product with neuronal protection, has a chemical structure similar to that of RES and plays an important role in nervous system diseases. Evidence suggests that PTE can attenuate cerebral ischemia reperfusion injury by inhibiting oxidative stress and neuronal apoptosis (Zhou et al., 2015), modulating microglial activation (Liu J. et al., 2020), and suppressing cyclooxygenase-2 (COX2) (Yan et al., 2021). It is reported that PTE alleviates Aβ_1–42_-induced cognitive dysfunction by inhibiting oxidative stress in SH-SY5Y cells (Xu et al., 2020) and regulating the phosphatidylinositol 3-kinase (PI3K)/AKT pathway in PC12 cells (Fu et al., 2016). However, research of PTE on AD is less comprehensive and in-depth. Therefore, the mechanism in AD needs to be further elucidated and improved.

In this study, behavioral tests are conducted to assess and investigate the effects of the two compounds, PTE and RES, in learning and memory. We demonstrated that both PTE and RES had effects on improving learning–memory deficits. However, PTE was more comprehensive in enhancing imaginal memory, working memory, and spatial memory. The reason for better pharmacological activities may be that PTE has a much higher bioavailability and liposolubility than RES (Kapetanovic et al., 2011; Chang et al., 2012; Liu Y. et al., 2020).

Neurons have been involved in cognitive processes and are the basis for learning–memory formation. Both *in vivo* and *in vitro* studies revealed that treatment with PTE significantly increased the number of neurons. These neuroprotective effects of PTE may indicate its potential value in the therapy of AD-related learning–memory deficits. Synaptic formation between neurons plays a vital role in the process of brain development, and the abnormalities of synaptic structure can lead to dysfunction in the brain neural circuitry (Jin et al., 2019). The vital plasticity-related proteins SYN-1 and PSD-95 have fundamental roles in the organization of the signal transduction at synapses (Savioz et al., 2014). Despite showing the beneficial roles of PTE in neurodegenerative diseases, the molecular mechanism associated with neuroplasticity is not fully understood in AD. It has been reported that PTE inhibits Aβ_1-42_-induced neuro-inflammatory in microglia via inactivating NLRP3/caspase-1 (Li et al., 2018). Herein, we found that PTE improved the synaptic structure and increased the expression of SYN-1, PSD-95, and NeuN in Aβ_25-35_-induced neuronal injury. Meanwhile, *in vitro* study showed that PTE improved the structure of dendrites. Mitochondria are involved in neuronal plasticity and provide ATP for processes involved in neurite outgrowth (Todorova and Blokland, 2017). Impairment in the mitochondria of neurons is one of the earliest events before clinical diagnosis during the pathological progression of AD (Mi et al., 2020). We found that mitochondrial dysfunction-induced apoptosis was related to the direct effect of Aβ_25–35_. PTE improved the mitochondrial structure and reduced apoptosis. These findings indicate that inhibition of mitochondria-dependent apoptosis by PTE exhibits neuroprotective effects in AD mice.

A previous study has demonstrated that RES, which has a chemical structure similar to PTE, is an activator of SIRT1 (Lee et al., 2019). The inhibition of mitochondria-dependent apoptosis by activating the antioxidant enzymes can reduce neurotoxicity (Yoo et al., 2017; Li et al., 2019). Considering that the SIRT1/Nrf2-related pathway modulates the expression of the antioxidant enzymes (Huang et al., 2018), we evaluated the roles of PTE in this process. In the present study, PTE increased the expression of SIRT1/Nrf2 and the level of SOD in Aβ_25-35_-induced neuronal injury. SIRT1 inhibitor EX527 blocked PTE from producing antiapoptosis in Aβ_25–35_-treated primary neurons. It is suggested that an important mechanism for neuroprotection of PTE may be due to the activation of a SIRT1/Nrf2-related antioxidant effect to inhibit apoptosis in neurons. It is known that microglia are the primary cells in the central nervous system and act as key players in neurodegenerative diseases. PTE is proved to alleviate neuronal apoptosis and oxidative injury by regulating SIRT1 expression in microglia-mediated inflammatory response (Zhu et al., 2020). In our study, PTE exerts neuronal protection via the activation of the SIRT1/Nrf2-related antioxidant effect to inhibit apoptosis. However, the relationship of PTE in regulating microglia and neurons is not clear; therefore, further study is required to improve the mechanisms of PTE.

In conclusion, our study demonstrated that compared with RES, the same dose of PTE could more comprehensively attenuate behavioral deficits induced by Aβ_25–35_. The possible mechanism could be related to inhibiting mitochondria-dependent apoptosis to improve neuronal plasticity through the SIRT1/Nrf2-related antioxidant effect. These results reveal that PTE may be a potential candidate for the treatment of AD.

## Data Availability

The raw data supporting the conclusion of this article will be made available by the authors, without undue reservation.
